# The 'PUCE CAFE' Project: the First 15K Coffee Microarray, a New Tool for Discovering Candidate Genes correlated to Agronomic and Quality Traits

**DOI:** 10.1186/1471-2164-12-5

**Published:** 2011-01-05

**Authors:** Isabelle Privat, Amélie Bardil, Aureliano Bombarely Gomez, Dany Severac, Christelle Dantec, Ivanna Fuentes, Lukas Mueller, Thierry Joët, David Pot, Séverine Foucrier, Stéphane Dussert, Thierry Leroy, Laurent Journot, Alexandre de Kochko, Claudine Campa, Marie-Christine Combes, Philippe Lashermes, Benoit Bertrand

**Affiliations:** 1 Nestlé R&D Tours, 101 Avenue Gustave Eiffel, Notre Dame d'Oé, BP 49716, 37097 Tours Cedex 2, France; 2 Montpellier Genomix, IGF, 141 rue de la Cardonille, 34094 Montpellier Cedex 05, France; 3 Boyce Thompson Institute for Plant Research, Tower Road, Ithaca, New York 14853-1801, USA; 4 IRD-CIRAD, UMR RPB, BP 64501, 34394 Montpellier, France; 5 IRD, UMR DIAPC, BP 64501, 34394 Montpellier, France; 6 CIRAD avenue Agropolis, 34398 Montpellier Cedex 5, France

## Abstract

**Background:**

Understanding the genetic elements that contribute to key aspects of coffee biology will have an impact on future agronomical improvements for this economically important tree. During the past years, EST collections were generated in Coffee, opening the possibility to create new tools for functional genomics.

**Results:**

The "PUCE CAFE" Project, organized by the scientific consortium NESTLE/IRD/CIRAD, has developed an oligo-based microarray using 15,721 unigenes derived from published coffee EST sequences mostly obtained from different stages of fruit development and leaves in *Coffea Canephora *(Robusta). Hybridizations for two independent experiments served to compare global gene expression profiles in three types of tissue matter (mature beans, leaves and flowers) in *C. canephora *as well as in the leaves of three different coffee species (*C. canephora*, *C. eugenoides *and *C. arabica*). Microarray construction, statistical analyses and validation by Q-PCR analysis are presented in this study.

**Conclusion:**

We have generated the first 15 K coffee array during this PUCE CAFE project, granted by Génoplante (the French consortium for plant genomics). This new tool will help study functional genomics in a wide range of experiments on various plant tissues, such as analyzing bean maturation or resistance to pathogens or drought. Furthermore, the use of this array has proven to be valid in different coffee species (diploid or tetraploid), drastically enlarging its impact for high-throughput gene expression in the community of coffee research.

## Background

In recent years, microarray technology has demonstrated the power of the high-throughput study of gene expression in unravelling key processes in plant biology [1-3]. Microarrays have become especially relevant for species where little genome information is available and where intensive laboratory work is necessary to gain insight into a particular biological process, as well as to identify candidate target genes for future breeding programs [4,5].

The genus Coffea (Rubiaceae family) encompasses approximately 100 species, all of which are native to the African continent, Madagascar and the Mascarene Islands [6]. Two of these species *Coffea canephora *(robusta) and *Coffea arabica*, are widely cultivated for the production of coffee beverages. The former is diploid (2n = 2× = 22) and allogamous, the latter, allotetraploid (2n = 4× = 44) and preferentially autogamous. Approximately 60% of the world coffee production comes from *C. arabica *versus 40% for *C. canephora*. In terms of cup quality, consumers appreciate *C. arabica *(Arabica) more due to its taste, which is less bitter and more flavourful compared with *C. canephora *(Robusta). While it is not widely known, coffee is one of the most valuable international exchange commodities in agricultural trade. This is reflected in the fact that raw coffee values rank fourth on the international stock market only after wheat, sugar, and soya [7]. Furthermore, over 25 million people worldwide are linked to coffee cultivation and processing. Despite these economic aspects, coffee research suffers from a lack of both scientific and financial investment. Also, coffee is a perennial plant which only begins to bear seed after about five years, which makes genetic studies more complicated and time-consuming. While some genomic information is publicly available for coffee (e.g., an expressed sequence tag (EST) database), it lags far behind what is available for many other agricultural species. As a result, coffee researchers have only limited access to the plethora of genomic resources available for most major crop species.

During the past few years, aiming to develop genomic tools to assist future coffee research, various scientific groups have produced large scale sets of *Coffea *EST sequences. However, the number of publicly available ESTs remains low because many of the sequences discovered fall under the domain of private property and are not published. At the time when the PUCE CAFE Project began, two large coffee EST databases were available: the NESTLE/Cornell and IRD databases with respectively 62,877 and 8782 sequences. Those sequences were mainly cDNA derived from leaves, fruit (whole cherries), pericarp and beans at different stages of maturation in *Coffea canephora *(robusta) [8,9]. The research aimed to catalogue as many genes as possible which are involved in the bean-filling period of fruit development in order to better understand the final composition of the beans which constitute the commercial product. The purpose of the PUCE CAFE Project was to develop a long oligonucleotide array based on available sequences and thus to use this new tool to perform large-scale transcriptomic analyses in different areas such as bean/fruit development, polyploidy or drought resistance in *Coffea canephora *or *Coffea arabica*. To assess its utility, we ran a comparison between three different tissues, i.e. mature beans, flowers and fully-expanded leaves, in *Coffea arabica *in order to catalogue genes specifically expressed in each tissue. We analyzed in particular the genes involved in fatty acid synthesis and storage proteins and compared our results with those in recent publications on *Coffea *[10] and also with exalbuminous bean species. Then we tested the usability of our 15k microarray for three coffee species.

## Methods

### The Coffee Gene Assembly (Build II)

To create the SGN Coffee Unigene Build II http://solgenomics.net/, 71,659 EST (Expressed Sequence Tag) chromatograms were processed from the following *C. canephora *sequence libraries: cccl (coffee leaf, 11,655 chromatograms), cccp (coffee pericarp, 10,849 chromatograms), cccs18w (coffee early-stage bean, 1,972 chromatograms), cccs30w (coffee middle-stage bean, 15,318 chromatograms), cccs42w (coffee late-stage bean, 42 weeks after pollination, 469 chromatograms), cccs46w (coffee late-stage bean, 46 weeks after pollination, 10,907 chromatograms), cccwc22w (coffee early-stage whole fruit, 11,660 chromatograms), irdccf (IRD coffee cherry in various developmental stages, 5,089 chromatograms), irdccl (IRD, young leaves, 3,693 chromatograms) and nDav1 (Nestle Dav1, 47 chromatograms), using PHRED software http://www.phrap.org/phredphrapconsed.html[11]. The sequences were processed to remove vector, adaptors and low complexity sequences using an SGN-developed Perl script). Chimeric sequences were screened by processing the BLAST results [12] using *Arabidopsis thaliana *ftp://ftp.arabidopsis.org/home/tair/Sequences/ as reference dataset and a SGN Perl script. A total of 55,539 sequences passed the filter tests and were used in the assembly. The unigene assembly was created in two steps. First, using a self-BLAST and an SGN Perl script (precluster.pl), we implemented a pre-clustering phase of the EST sequences with a minimum identity percentage of 90% and a minimum alignment length of 30 bp. Secondly, we used CAP3 software http://seq.cs.iastate.edu/[13] for each cluster with the following parameters: -e 5000 -p 90 -d 10,000 -b 60. The -e, -d and -b options were set so that the assembler would disregard them or minimize their effect. The -p option increased the sequence identity necessary with overlaps to 90 from a default of 75, and thus was found to be lacking in stringency.

Concerning unigene annotations, we first made a homology search using the BLAST program against GenBank ftp://ftp.ncbi.nih.gov/genbank/ and *Arabidopsis thaliana *ftp://ftp.arabidopsis.org/home/tair/Sequences/ datasets, setting an e-value of 1e-10 as the cutoff value. Secondly, we implemented a prediction of protein sequences based on unigene sequences using ESTScan software http://estscan.sourceforge.net/[14] and an SGN Perl script (longest6frame.pl), which simply determines the longest open reading frame and translates it into a protein sequence. Thirdly, we set up a protein domain homology search on predicted protein sequences using InterProscan software http://www.ebi.ac.uk/Tools/InterProScan/[15]. All the information concerning the different scripts used to perform EST assembly are available on https://github.com/solgenomics/sgn-home/tree/master/aure/scripts/old_sgn_transcript/.

### Long Oligonucleotide Microarray Design and Synthesis

The *Coffea canephora *long oligonucleotide set was designed and synthesized by Operon (Cologne, Germany) based on the SGN Coffee Build II (15,721 unigenes; http://solgenomics.net/). An amino linker was attached to the 5'-end of each oligonucleotide. The oligonucleotides, selected to limit secondary structure, have a melting temperature of 67 ± 3°C, length 65 ± 5 bases, GC content 43 ± 5%. More than 98% of the oligonucleotides were within 1000 bases from the 3'-end of the available gene sequence. For 195 unigenes, no adequate oligonucleotide could be designed and therefore correspond to "missing genes" (Additional File 1). BLAST alignments were performed to identify oligonucleotides that could cross-hybridize with other sequences of the SGN Coffee Build II. Finally out of 15,522 oligonucleotides designed, there are 371 oligonucleotides which have > 70% of overall identity to another unigene and have a contiguous identical length of over 20 nt common to another unigene (Additional File 2).

### Plant Material and RNA Extraction

In a first experiment (Experiment 1), we compared three tissues, namely fully-expanded leaves, open flowers and mature beans. They were collected from *C. arabica L. cv. Caturra T 2308 *grown in greenhouse conditions in Tours, France.

In a second experiment (Experiment 2), we compared fully-mature leaves of three species (namely *C. arabica, C. canephora and C. eugenioïdes*) to determine if our microarray could be used for different coffea species. *C. arabica *was represented by the *cv*. 'Java' issued from the Arabica woodland Ethiopan pool and by one genotype representing the Arabic-cultivated pool. *C. canephora *was represented by the *cv*. 'Nemaya' derived from the cross of two Congolese genotypes. Finally, *C. eugenioides *was represented by several genotypes, collected in Kenya at the Mount Elgon. The coffee seedlings were grown in a greenhouse with natural daylight and a constant temperature of 24° C and watered as necessary. After 120 days, the plants were transferred for an additional 60 days to a phytotron chamber (CRYONEXT, France, model RTH 1200L). The standard conditions in the phytotron were 12-hr light (600 μmol.m^-2^s^-1^, 26° C), 12-hr dark (22° C), with 80% to 99% relative humidity. In each growth chamber three plants for each species were cultivated. Each plant represented one replicate. Two fully-developed leaves were collected from each plant (i.e. two leaves/replicate) at noon (6-8 hours after lights on) and then flash-frozen in liquid nitrogen.

Tissues were ground into a powder and total RNA was extracted using the RNeasy Plant Mini Kit (Qiagen; Valencia, Cal., USA), then treated with DNase following the manufacturer's instructions. Total RNAs were finally eluted from the columns with RNase-free water (2 × 30 μL). For each tissue, three independent RNA extractions were performed. All RNA samples were analyzed by formaldehyde agarose gel electrophoresis to assess their integrity. To test for contamination by polyphenols, carbohydrates and proteins, a NanoDrop ND-1000 spectrophotometer (NanoDrop Technologies; Wilmington, Delaware, USA) was used. Only RNA samples with OD 260/280 > 1.8 and OD 260/230 > 2 were used for further analysis.

### RNA Labelling

For the preparation of the labelled Cy3- and Cy5- aRNA target, one microgram of the total RNA samples were amplified and labelled using the Amino Allyl Message Amp II aRNA Amplification Kit (Ambion; Austin, Texas, USA), according to the manufacturer's instructions.

### Microarray Printing

The synthesized oligonucleotides were arranged in 384-well plates, and dissolved at 20 μM in a phosphate buffer (150 mM, pH 8.5). The oligonucleotide probes were printed on reflective epoxysilane-coated slides (Amplislide, Genewave, Ecole Polytechnique, France) using a Lucidea Array printer (GE HealthCare, St. Catharines, Ontario). The oligonucleotides library also included sets of positive and negative control points that were used for verifying, for example, the quality of the microarray and mRNA, the sensitivity and linearity of the signal, or the consistency of the assay. In addition, the expected dye ratios were determined and the differences in signal intensities due to the differences in dye incorporation and quantum yield were estimated.

### Hybridization

Prior to hybridization, oligonucleotides were cross linked to the slides by UV irradiation at 100 mj and the excess was removed from the arrays by washing them twice in one minute in 0.2% sodium dodecyl sulphate (SDS). Arrays were then washed twice in distilled water. The two labelled aRNA were added to Microarray Hybridization Buffer Version 2 (GE HealthCare, St Catharines, Ontario) in a final concentration of 50% formamide, denaturated at 95° C for three minutes and applied to the microarrays in individual chambers of an automated slide processor (GE HealthCare, St Catharines, Ontario). Hybridization was carried out at 37° C for 12 hours. Hybridized slides were washed at 37° C successively with 1× Saline Sodium Citrate, 0.2% SDS for 10 minutes, twice with 0.1× SSC, 0.2% SDS for 10 min, with 0.1× SSC for one minute and with isopropanol before air drying.

### Data Acquisition

Microarrays were immediately scanned at 10 μm resolution in both Cy3 and Cy5 channels with GenePix 4200AL Scanner (Molecular Devices, Silicon Valley, Cal., USA) with variable photo multiplier tube (PMT) settings to obtain maximal signal intensities with <0.1% probe saturation. ArrayVision^® ^software (GE HealthCare, St Catharines, Ontario) was used for feature extraction. Spots with high local background or contamination fluorescence were flagged manually. A local background was calculated for each spot as the median values of the fluorescence intensities of four squares surrounding the spot.

### Real-time PCR

We carried out reverse transcription of total RNA using random hexamer oligonucleotides and SuperScript II Kit (Invitrogen, Carlsbad, Cal., USA) according to the manufacturer's instructions. Real-time PCR was performed on a LightCycler^® ^480 equipped with a 384-well block using the LightCycler^® ^480 SYBR Green I Master Mix (Roche Diagnostics, Indianapolis, Ind., USA) according to the manufacturer's instructions. The primer sequences used for the determination of gene expression levels are given in Additional File 3. The selection of appropriate housekeeping genes was performed using geNorm [16]. The level of expression of each gene X was normalized to the geometric mean of the expression levels of 3 reference genes (Spermidine synthase 1, Cyclophilin and Actin-11), according to the formula

$$\frac{X}{\sqrt[{3}]{R1\times R2\times R3}}=2^{\left(Ct\left(X\right)-\left(\frac{Ct\left(R1\right)+Ct\left(R2\right)+Ct\left(R3\right)}{3}\right)\right)}$$

where Ct is the threshold cycle and R1, R2, R3 are the 3 reference genes.

Additional information concerning the Q-PCR experiment can be found in the MIQE document (Minimum Information for Publication of Quantitative Real-Time PCR Experiments) (See Additional File 4).

### Experimental Design and Data Analysis

For the first experiment, for qRT-PCR as for microarray, three biological replicates were made for each tissue analyzed (i.e. leaves, flowers and mature beans). The following comparisons were made: Bean-Flower, Leaf-Flower and Leaf-Bean. In all, we performed microarray analyses on 18 slides [3 (replicates) × 2 (dyes) × 3 (organs)]. For qRT-PCR we performed 3 technical replicates × 3 biological replicates × 3 organs for 108 genes and three reference genes (R1, R2 and R3) for each tissue.

For the second experiment, three biological replicates were done, each of one containing two leaves. In total we used 36 slides [3 (replicates) × 2 (dye) × 6 (comparisons)]. All microarray analyses were performed using Bioconductor http://www.bioconductor.org, the open development software project for the analysis and comprehension of genomic data.

### Preprocessing

A quality analysis was made by generating image plots (MA-plots, boxplot, visualization of the array). No background correction was performed. Few spots were flagged and controls were removed for the normalization. Loess normalization was performed for each microarray to correct the dye effect and technical bias. Then the microarray data were filtered keeping the spot intensity above a median of 90 percent of the control spots (Empty/Negative Control - *NC*) plus twice the deviation standard, applied to both channels (Red and Green).

### Statistical Analysis

Two tests of differential expression were conducted simultaneously: the first test fit a linear model for the expression data for each gene by using the Limma Package (Linear Models for Microarray Data) [17], the second test SAM used repeated permutations of the data to determine significant genes [18]. It was conducted with the Siggenes package from Bioconductor. Multiple testing adjustments were performed by using a false discovery rate approach [19]. These two analyses allowed us to rank significantly expressed genes. The Bioarray Software Environment (BASE - [20]) (local installation: http://baseprod.igf.cnrs.fr/index.phtml) was used to visualize the differential expression for each gene.

### Reproducibility of Biological Replicates and Specificity of the Microarray

For the first experiment, coefficients of variation were calculated for the mean signal intensity for the 6 slides (i.e, two dyes × three biological replicates). Using the procedure rank in SAS 9.2 (SAS Institute, Cary, NC), each CV received a rank according to the size of the CV value. These rank values were then expressed on a 100-based scale.

The effect of sample size on the power of statistical tests for different CVs was estimated using the sample size estimate procedure for a two-sample t-test in SAS 9.2 with α = 0.01, group 1 mean = 1, and group 2 mean = 1.5 or 2.5. The effect of the CV on the minimally detectable expression ratio (threshold expression ratio) was iteratively estimated for a power of 0.9 and α = 0.01 using the sample size estimate procedure for a two-sample t-test.

To validate the expression changes found in Microarray Experiment 1, transcript levels of 108 genes and three reference genes were quantified by Q-PCR (with validated primers [10]). Results obtained by both techniques (microarray and Q-PCR) (Additional File 3) were compared by calculating the Pearson correlation coefficients (SAS 9.2). For the microarray, the data input into the correlation analysis was the Log_2 _ratio value of the weighted average for each gene on the composite array representing all replicates. For qRT-PCR, we used the mean Log_2 _ratio value representing all replicate plants. All correlation analyses carried an alpha value of 0.01 and were performed using SAS 9.2.

For the second experiment, background noise was defined from the intensity of "negative" controls (NC). The median of the negative controls was calculated. A gene was considered expressed if the intensity exceeded twice the standard deviation. If a gene is significantly expressed during a comparison (6 hybridizations), its signal should be superior to the highest background noise in each hybridization (maximum 6 times). We chose to fix this threshold to 5, and for each species we screened the number of genes that reached or surpassed this value (Additional File 5).

A hierarchical tree-clustering support method was performed using TMeV 4.0 software from TIGR. The Euclidean distance was used as a measure of similarity or distance between hybridizations. As a rule, the Average-Link Method was used for linking clusters. With this method, distance calculations are based on pairs of clusters: taking the average between the distance of objects from the first cluster and of objects from the second cluster. The averages are performed for all pairs to determine the actual distance between clusters.

Venn diagrams were generated using the online Venny tool http://bioinfogp.cnb.csic.es/tools/venny/index.html.

### Gene Ontology Functional Enrichment Analysis

Computational annotation was also performed using Blast2GO software v2.4.4. (http://www.blast2go.org website) [21]. The annotation step was performed using the BlastX algorithm, the NCBI nr database and a Blast expectation value threshold of 1E-3. The Blast2GO tool was then used to obtain GO information from retrieved database matches. Mapping of all sequences was performed using default parameters. An InterPro Scan was also performed to find functional patterns and related GO terms by using the specific tool implemented in the Blast2GO software with the default parameters. Finally, an enrichment analysis was completed for the sets of up-regulated unigenes in each of the three tissues studied using the corresponding Blast2GO module, which is based on Fisher's Exact Test and FDR statistics. Additional File 6 shows GO terms showing a significantly higher or lower frequency in tissue-specific unigene sets in comparison with the full set of unigenes of the PUCE CAFE array.

### Availability of the Microarray Data

Microarray data are publicly available at http://www.ncbi.nlm.nih.gov/geo/. The GEO accession number is GSE24754 for Experiment 1 and GSE24682 for Experiment 2. The array is referenced as GPL10928.

## Results and Discussion

### Chip Quality

To estimate the quality of the work performed by the MGX platform (Montpellier, France), one validation experiment (Experiment 1) was implemented. Expression was compared in three tissues, namely fully-expanded leaves (L), open flowers (F) and mature bean (B) in *Coffea Arabica*. Three biological replicates were used for each tissue. The following comparisons were studied: Bean-Flower, Leaf-Flower and Leaf-Bean.

**Figure 1:**
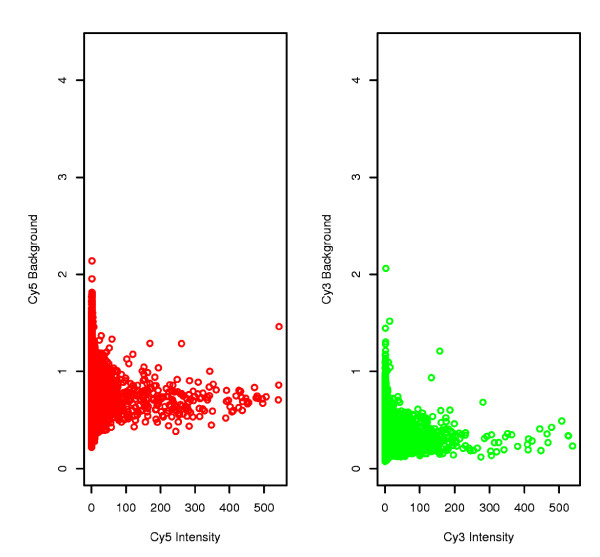
**Plots for each Channel of Background Intensity versus Intensity**. The background is uncorrelated and does not increase along with the intensity. Flower *vs*. leaf comparison data are presented here.

Raw quantification and background noise values were represented for each chip (data not shown). Visually, the flags indicated invalidated spots. In this way it was possible to visualize whether there were any particular artefacts on a slide, due for example to washing impurities or to the presence of dust. The distribution of raw intensities, background noise and log-ratios were uniform. Very few spots were flagged (Table 1) and background noise was low and virtually constant when signal intensity increased (Figure 1) indicating that the chips were of very good quality.

**Table 1:** In Experiment 1, for each comparison (six slides) 16,512 spots were examined.

Comparisons	Number of Spots invalidated by visual examination (Flags)
Leaf/bean	16
Leaf/flower	34
Bean/flower	7

The number of genes invalidated by visual examination is indicated.

### Signal Distribution for each Hybridization and Data Standardization

Gene expression was compared as a function of the dye (Cy3 or Cy5). Background noise was defined from the intensity of "negative" controls (NC). The median of the negative controls was calculated. A gene was considered expressed if the intensity exceeded twice the standard deviation. We compared efficiency for Cy3 and for Cy5 incorporation. The dye bias was greater for low-value signals. Of 15,998 genes, 40.76% were always significantly expressed compared to background noise for the red dye and 44.70% for the green dye. This bias was largely corrected by standardizing the data using the Loess regression method.

**Figure 2:**
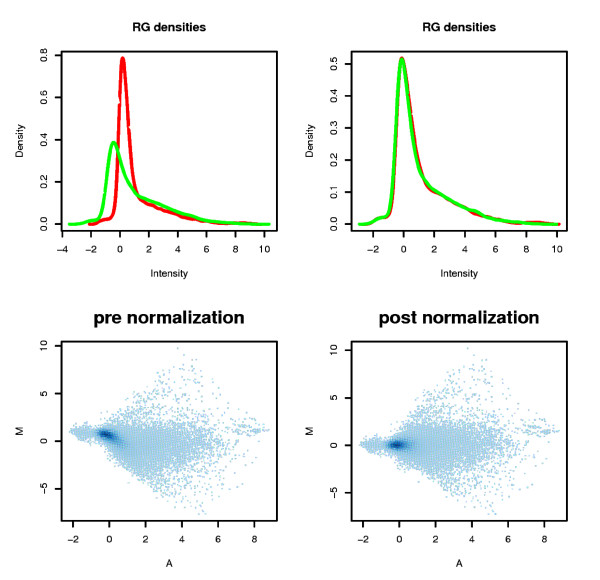
**Density Plot and MA-plot for Both Channels Before and After Normalization**. *Density plot*: green and red curves represent the densities of the intensity of the green and the red channel. After normalization, the curves are similar. *MA-plot*: After normalization, the data fall to a straight horizontal line along 0; before normalization, there is a slight upward curve. Data from leaf *vs*. bean comparison (Hybridization No. 1, Experiment 1) are presented in this example.

After standardization, the curves for the Cy3 and Cy5 signals were superposed upon the density graphs (Figure 2). The MA-plot scatterplots did not display any striking differences as most of the technical biases were reduced by using automated protocol as well as specific slides for reducing differences due to dye incorporation.

**Figure 3:**
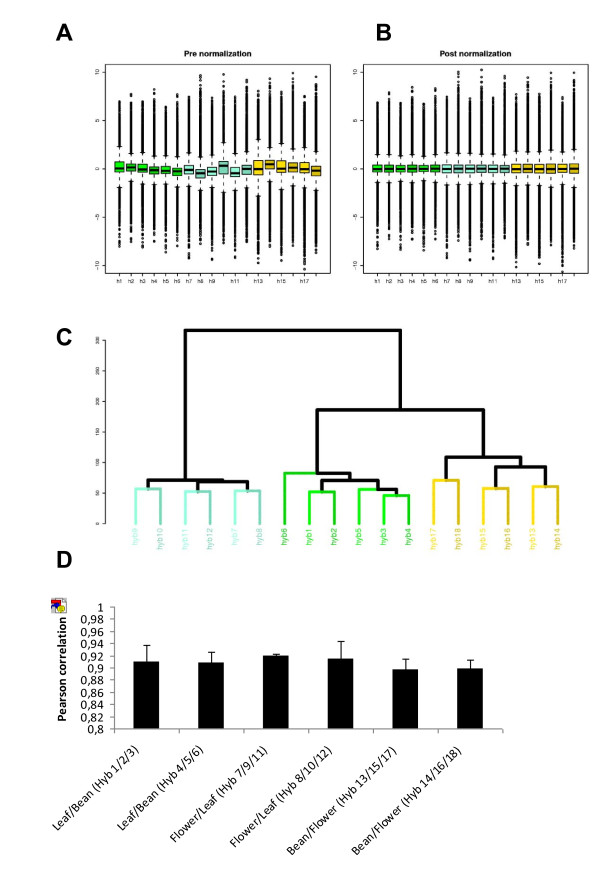
**Quality Analyses between Biological and Technical Hybridization Replicates for Experiment 1 (Bean, Flower and Leaf)**. Hybridizations 1-6 correspond to Leaf-Bean comparisons. Hybridizations 7-12 correspond to Flower-Leaf comparisons and hybridizations, and 13-18 correspond to Bean-Flower comparisons. The box-plots allow us to compare the log2 (ratio) distribution of all the hybridizationsbefore (A) and after (B) normalization. The distribution of log2 ratios for all the comparisons is shown on one plot. The baseline is set to a similar raw expression level, allowing the inter-chips comparison. (C) Hierarchical clustering of samples using Euclidean Distance on normalization data. The samples cluster primarily by replicates. In blue is the flower-leaf comparison; in green, the leaf-bean comparison and in brown, the bean-flower comparison. (D) Person correlation at gene-level for all the probes in the replicates of the microarray. All the replicates showed a correlation value greater than p > 0.89 thus showing a high level of similarity.

Box-plots of both pre- and post-normalization (Figure 3A and 3B) confirmed that our data were successfully normalized. Data quality was assessed by comparing the signal intensity data from each array to that obtained from the technical or biological replicates. Pearson correlation between replicates was calculated for every gene in all the arrays, resulting in a very high correlation level, with a coefficient of >0.89 for every independent experiment in a pairwise comparison (Figure 3D). This high coefficient is indicative of the precision level in which the microarray is able to process transcriptomic data reliability.

A support tree-clustering method with bootstrapping using expression data was performed to statistically validate the tool. Figure 3C showed a high level of similarity between the replicates.

### Reproducibility of Biological Replicates

The variability between expression profiles derived from the two dyes and the three biological replicates was estimated. For each experiment we calculated the coefficient of variation (CV) for the mean signal intensities for six slides (i.e. 2 dyes × 3 biological replicates). Ninety percent of the spots on the arrays could be determined with a CV of less than 42% (Table 2). These values are consistent with those obtained previously [22]. The CV (%) was then plotted against the relative rank of the CV (Additional file 7). Based on this estimated variance, a power analysis was performed for a two-sample t- test (Additional file 8).

**Table 2:** Mean, Median and P90 of the Coefficients of Variation (CV) of the Fluorescence Signal Intensity

Comparison	Tissue	Mean	Median	P90
**Leaf/bean**	Leaf	27.31	24.15	41.58
	Bean	25.66	21.82	38.93
**Leaf/flower**	Leaf	23.38	21.76	35.68
	Flower	22.92	20.46	32.59
**Bean/flower**	Bean	23.28	20.58	37.56
	Flower	19.90	17.96	32.26

With a CV of 35%, a sample size of six slides can detect a 2-fold change in gene expression with a power of 90%, with a Type I error rate of 10%. The detection of 1.5-fold changes with a Type I error rate of 0.1, a power of 90% and sample size of six slides requires the CV to be below 20%. We therefore concluded that a sample size of six slides would allow acceptable control of both Type I and Type II errors.

### Analysis of Differential Expressions

**Figure 4:**
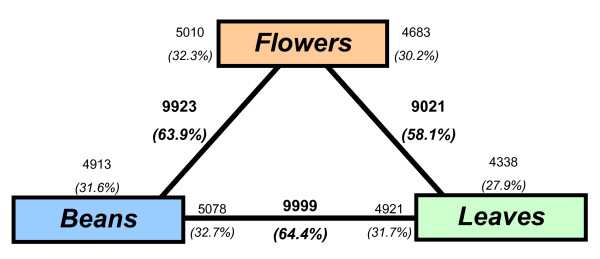
**Transcriptome divergence between the three tissues (flower/leaf/mature bean)**. The total number and fraction of genes diagnosed as differentially expressed in each contrast are indicated in bold text. Also shown for each contrast is the partitioning of the total number of differentially expressed genes in the direction of up-regulation; these numbers are indicated in non-bold text. For example, 9923 genes are indicated as being differentially expressed between flowers and leaves. Of these, 5010 (32.3%) were up-regulated in flowers, and 4913 (31.6%) were up-regulated in beans. Around 58-65% of the 15,522 unigenes were differentially expressed between the three tissues.

**Figure 5:**
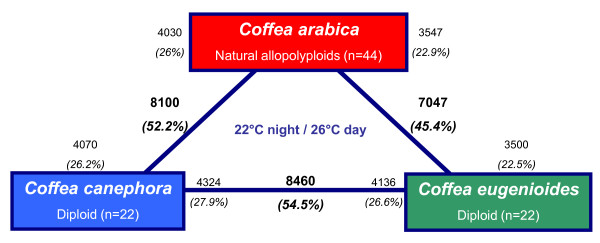
**Transcriptome divergence between *C. arabica*, *C. canephora *and *C. eugenoïdes***. Bold text indicates the total number and fraction of genes that were defined as differentially expressed between each comparison. Non-bold text indicates the total number and fraction of genes that were in the direction of up-regulation. For example, 8100 (52.2%) genes were indicated as being differentially expressed between *C. canephora *and *C. arabica*. Of these, 4030 (26%) were up-regulated in *C. arabica*, and 4070 (26.2%) were up-regulated in *C. canephora*.

**Figure 6:**
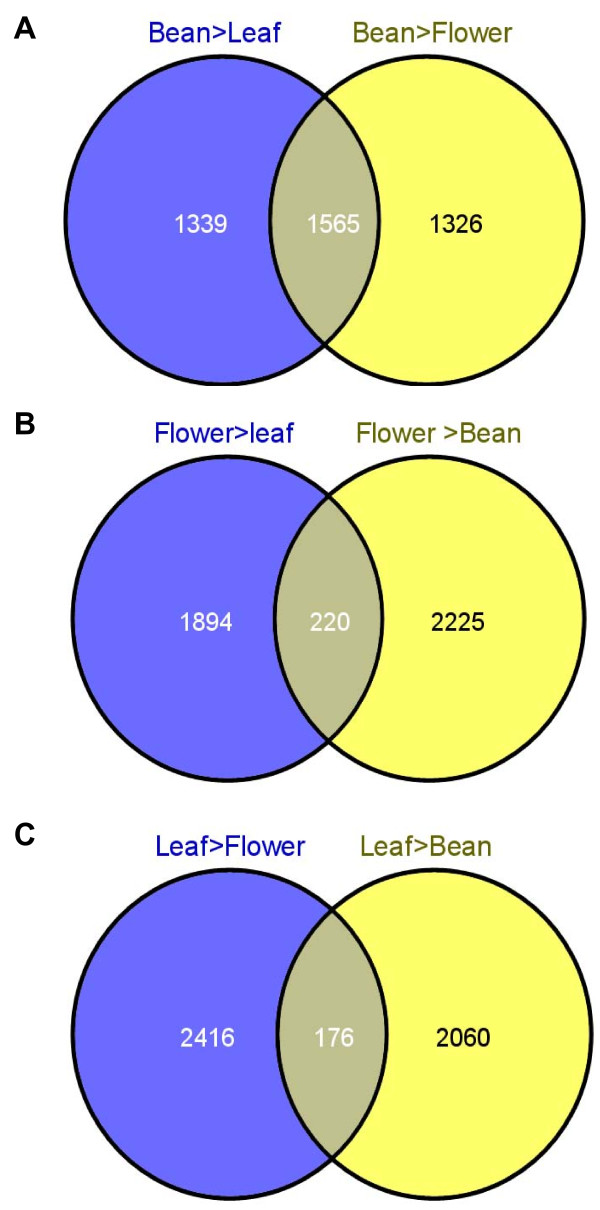
**Venn Diagrams indicated genes that are over-expressed specifically in each tissue (Bean, Flower and Leaf)**. The Venn Diagram using all deregulated genes identified by Limma Analysis (P ≤ 0.01) for each comparison identified genes that are over-expressed specifically in each tissue compared to the two others. A, B and C. 1565, 220 and 176 genes are specifically over-expressed in bean, flower and leaf respectively.

Two statistical analyses were performed on normalized data from Experiments 1 and 2, one by the Limma Method (linear model), the other by the SAM Method (significance analysis for microarray) with two thresholds P = 0.01 and P = 0.05. For each comparison, lists of differentially-expressed genes were generated. One list was derived from the "Limma" analysis, the other from the "SAM" analysis. The files are available on BASE http://baseprod.igf.cnrs.fr/index.phtml. The Limma Method was more restrictive than the SAM Method, so the number of genes determined as being significantly differentially expressed was therefore smaller. Nevertheless, the totality of these genes was also detected by the SAM Method in the three comparisons conducted. These genes were therefore validated by two independent methods (results not shown). The lists resulting from the SAM 0.01 analysis were used to compare differentially-expressed genes for each comparison (Figure 4 for Experiment 1 and Figure 5 for Experiment 2). The lists resulting from the Limma P = 0.01 analysis was used to identify over-expressed genes in one specific tissue (bean flower or leaf) (Figure 6 Additional file 9).

### Comparison of the Three Tissues (Mature Bean, Flower, Leaf)

The number of genes that were differential and significantly expressed when comparing different tissues was between 9,021 and 9,999 genes (Figure 4), i.e. between 58% and 64% of genes spotted on the array. For each comparison, the percentages of up-regulated genes varied between 30 and 33%. The three tissues therefore behaved globally in an identical manner.

### Correlation between qRT-PCR and Microarray Results for 111 Genes

Consistent with the previous results [23], fold change results determined by qRT-PCR were significantly greater than fold change assessed for the same genes by microarray analysis. Correlations for the data sets (i.e., bean/flower, flower/leaf, leaf/bean) ranged from 0.78-0.81 (p < 0.01) for the 108 genes analyzed (Additional file 3). The direction of change was similar for both qRT-PCR and microarray for 70-75% of the genes analyzed. Furthermore, when we correlated only the significantly-expressed genes (SAM 0.05) (> 1.5 fold change), the degree of correlation between microarray and qRT-PCR results was higher, ranging from 0.85-0.87 (p < 0.01) for 83 genes. The lack of congruence between both methods for genes exhibiting low levels of variation (< 1.5 fold change) has been commonly reported [24]. These results validate the implicit assumption that there is a good correlation between the microarray data and the mRNA levels in the tissue under investigation.

### Specifically Over-Expressed Genes in each Tissue (Mature Bean, Flower, Leaf)

Based on the lists of deregulated genes identified for each comparison (Limma Analysis; P = 0.01), Venn diagrams were constructed (Figure 6). Over-expressed genes in each specific tissue were so listed (Additional file 9). 1,565 genes were significantly over-expressed in the bean compared to the leaves and flowers (Figure 6A and Additional file 9). Likewise, 220 "flower-specific" (Figure 6B) and 176 "leaf-specific" (Figure 6C) genes were identified.

The number of genes which are over-expressed in the bean is significantly higher than those identified in flower and leaf. This observation is quite normal since the PUCE CAFE array is mainly based on genes expressed in the grain during fruit maturation.

This analysis is quite interesting and clearly identified the genes involved in different metabolic pathways specific to each organ. In order to shed light onto the processes involved under the conditions studied, we enriched the Gene Ontology (GO terms) among up-regulated genes in the three different organs. Additional file 6 shows GO terms showing a significantly higher or lower frequency in tissue-specific unigene sets in comparison with the full set of unigenes of the PUCE CAFE array.

### Validity of Microarray Results related to Biosynthesis Pathways of Lipids or Storage Proteins

To test the accuracy of the results obtained with the 15k coffee microarray a bit further, we compared expression patterns of a few genes involved in well-characterized biosynthetic pathways of lipids or storage proteins with those described in studies on *Coffea *and model plants. As storage tissue, the mature endosperm accumulates nutrient reserves (mainly cell-wall polysaccharides, sucrose, proteins and oils) which are mobilized by the embryo during germination and seedling growth. As expected, most of the genes involved in the accumulation of these storage compounds displayed enhanced transcriptional activity in the bean compared to leaves and flowers.

### Study of Different Genes Involved in the Biosynthesis of Lipids

In coffee leaves, linolenic acid (18:3) is the predominant FA (fatty acid) [25], whereas it represents only a small percentage of the total FA in beans [26]. Accordingly, the gene encoding the enzyme involved in converting linoleic acid to linolenic acid (ω-3 desaturase, *FAD8*) was significantly over-expressed in leaves compared to beans (Table 3). Similarly, although waxes can be detected in beans and flowers, these compounds predominantly accumulate in leaves. The first step of wax biosynthesis involves a β-Ketoacyl-CoA Synthase (KCS) activity, which initiates the biosynthesis of a very-long chain of fatty acids specific to waxes. Again, the microarray showed evidence of a higher KCS gene expression in leaves compared to other tissues (Table 3).

**Table 3:** Expression Patterns of a few Genes Involved in Well-Characterized Lipid Biosynthetic Pathways.

Gene	SGN Accession	Putative Function	E value	% Id	B/L Ratio	B/F Ratio	L/F Ratio	Tissue Specificity
***DGAT***	SGN-U349452	Acyl-CoA Diacylglycerol acyltransferase (At2g19450)	1E-103	78	4	8.7	2	Bean > L > F
***OLE-2***	SGN-U350187	Oleosin CcOLE-2 (AY841272)	0	100	9.6	9.1	1.3	Bean > L-F
***FAD8***	SGN-U349395	Plastidial Linoleate Desaturase FAD8 (At5g05580)	0	73	0.021	0.085	3.8	Leaf > F > B
***KCS***	SGN-U359520	-Ketoacyl-CoA Synthase (At1g68530)	2E-87	64	0.023	0.526	21	Leaf > F > B
***Fat B***	SGN-U350529	Acyl-ACP Thioesterase (At1g08510)	1E-153	78	1.1	0.196	0.213	Flower > L-B
***JMT***	SGN-U349158	SAM:jasmonic acid carboxyl methyltransferase (At1G19640)	3E-34	42	4.6	0.0025	0.0087	Flower > L-B

Comparison: bean to leaves (B/L), bean to flower (B/F), leaf to flower (L/F).

The acyl-ACP thioesterase (encoded by the *Fat B *gene) was described as the control point of the remarkably high palmitic acid content of Arabidopsis flowers in comparison with other organs [27]. A similar expression pattern was observed for the putative *FatB *gene in coffee, suggesting that the coffee flower could also be highly rich in this fatty acid. Finally, since coffee flowers are well known for their jasmine fragrance, we investigated the expression pattern of a putative *JMT *gene that encodes a jasmonate O-methyl transferase. Indeed, the volatile plant hormone jasmonate and methyl-jasmonate are also directly involved in flower fragrance [28]. As expected, *JMT *expression appeared to be highly specific in coffee flowers (Table 3).

Finally, the bean-specific expression of genes encoding DGAT (Table 3), the enzyme catalyzing the last step of triglyceride synthesis [29], and Oleosin-2, a structural component of oil bodies [30], provided that we had a good signature of storage lipid accumulation in the coffee bean.

### Study of Genes Encoding Main Proteins Stored in Mature Beans

The storage proteins in the bean constitute the major portion of the proteins found in ripe beans. The expression of these proteins is temporally regulated during the coffee cherry ripening period and is restricted to bean tissues such as cotyledons or endosperm [31]. The coffee storage protein 1 (*csp1*) mRNA encoding 11 S globulin is highly accumulated in ripe beans and poorly detected in leaves or flowers (Table 4) as shown in previous publications [10,32]. The coffee storage protein 2 (*csp2*) mRNA is also detected in the ripe bean but considerably less than *csp1*, suggesting that among the *csp *gene family a strong difference of expression can be observed from one member to another.

**Table 4:** Expression Patterns of a few Genes encoding Potential Storage Proteins.

Gene	SGN accession	Putative function	E value	% Id	B/L ratio	B/F ratio	L/F ratio	Tissue specificity
*csp1*	SGN-U350946	11 S plant bean storage protein *Coffea arabica *(Y16975)	1-e134	100	103	93	0.925	B > F-L
*csp2*	SGN-U347807	11 S plant bean storage protein (At2G28490)	1e-121	50	28.56	12.75	0.315	B>F > L
*CcLEAP2*	SGN-U350577	Late embryogenesis abundant protein (At1G52690)	1e-12	60	257	6.97	0.01	B>F > L
*CcLEAP3*	SGN-348605	Late embryogenesis abundant protein (At2G40170)	8e-24	73	592.76	339.53	0.38	B > F-L
*CcLEAP4*	SGN-347291	Late embryogenesis abundant protein (At4g02380)	2e-15	50	6.26	1.82	0.32	B>F > L

Comparison: bean to leaves (B/L), bean to flower (B/F), leaf to flower (L/F); csp (coffee storage protein); LEAP (late embryogenesis abundant protein).

The late embryogenesis abundant (LEA) proteins, a diverse class of highly abundant, heat-stable proteins, accumulate late in embryo maturation or in endosperm. This accumulation coincides with the acquisition of desiccation tolerance that occurs also during coffee bean ripening. These proteins can be detected in vegetative organs, especially under stress conditions such as cold, drought, or high salinity [33].

*CcLEAP2 *and *CcLEAP3 *are highly expressed in ripe beans. While *CcLEAP3 *is not detected in flowers and leaves (Table 4), *CcLEAP2 *is significantly expressed in flowers. *CcLEAP4 *is expressed significantly in the three tissues analyzed but its manifestation in beans is quite low compared to *CcLEAP2 *and *CcLEAP3*. This wide difference of expression is also largely observed in *Arabidopsis *[33] among the 51 LEA proteins identified in the genome, suggesting different functions for each member of this superfamily.

### Utility of the 15k Microarray for Different Coffee Species

Although the long oligonucleotides spotted on the 15K coffee microarray were defined from *Coffea canephora *EST sequences principally derived from genes expressed during coffee fruit development and leaves. We tested the microarray utility tool for two other coffee species, namely *C. arabica *and *C. eugenioides*. In these two species and in *C. canephora*, evolutionary (divergence) is quite recent (< 100 000 to 10 000 years) and their nucleotide divergence was recently estimated at below 5% [34]. Furthermore, *C. canephora *and *C. eugenioides *are considered to be the diploid parents of *C. arabica *(tetraploïd) [34].

Transcriptomic differences between the two Arabica genotypes (data not shown) appeared minor, so for the present study we considered the average response of both genotypes for the Arabica species. We observed that 8226, 8270 and 8530 genes were significantly expressed in comparison to the background noise in *C. Canephora*, *C. Arabica *and *C. Eugenoides *respectively (Additional File 5). These last results indicate that about 53% of the genes represented on the chip are specifically expressed in leaves. We also calculated that 97.4% of these expressed genes are common in the three species.

A large difference was observed between the transcriptomes of the parental diploids *C. eugenioïdes *and *C. canephora *as 54.5% of the 15,522 genes were differentially expressed (Figure 5). Among the differentially-expressed genes, equivalent proportions were up-regulated in each parent 27.9% for *C. canephora *versus 26.6% for *C. eugenioides *(Figure 5). A high fraction of genes was differentially expressed between *C. arabica *and their parents, between 7047 and 8100 genes were indicated as being differentially expressed in *C. arabica *and *C. eugenioïdes *and *C. canephora *respectively. Of these, 23 to 26% were up-regulated in *C. arabica *and 23-26% were up-regulated in the two diploid species (respectively *C. eugenioïdes *and *C. canephora*). Finally, a low variation (~ 5.4%) in percentages of differentially expressed genes was observed between the three comparisons.

It can be stated that our microarray tool may be used to analyze global expression not only in *Coffea canephora *but also in other important species such as *Coffea arabica *(which represents 70% of the coffee market) or wild species such as *Coffea eugenoïdes*.

## Conclusions

We present here the creation and validation of the first coffee oligonucleotide-based microarray tool for functional genomic studies in coffee. Our results reveal that this new tool applies to high-throughput gene expression analyses in various *Coffea *species. Furthermore, the use of the array has proven to be valid for genomic studies on different plant tissues. As proof of principle, we have reported changes in gene expression generated by this microarray in two independent experiments. The statistical analyses of our microarray data, the correct correlation between Q-PCR and the microarray data validate our chip. Overall the coffee microarray (designated as "PUCE CAFE") offers the possibility to carry out functional genomic studies in a wide variety of research areas such as plant development, biotic and abiotic stress response or fruit quality traits. This new tool will be valuable for researchers interested in *Coffea *transcriptomics and will be available through the MGX platform.

## Competing interests

The authors declare that they have no competing interests.

## Authors' contributions

IP and BB have contributed equally to this work. The project was coordinated by IP, PL and BB. All the authors have read the manuscript and agree with the contents.

## Supplementary Material

Additional file 1 **The Missing Genes**. List of 195 unigenes for which the design of specific oligonucleotides could not be achieved and that are therefore absent from the PUCE CAFE microarray. Click here for file

Additional file 2 **Oligonucleotides that may cross-hybridize with several sequences**. List of 371 oligonucleotides spotted on the microarray which have > 70% of overall identity to another unigene and have a contiguous identical length of more than 20 nt common to another unigene. Click here for file

Additional file 3 **Q-PCR and Microarray Data for 111 Genes of Interest Extracted from Experiment 1**. Microarray and Q-PCR were compared by calculating the Pearson correlation coefficients. For the microarray, the data input into the correlation analysis was the Log2 ratio value of the weighted average and for each gene on the composite array representing all replicates. For qRT-PCR, we used the mean Log2 ratio value representing all replicate plants. All correlation analyses carried an alpha value of 0.01 and were performed using SAS 9.2. The list of the primers and relative sequence used for the qPCR are indicted as well as the efficiency of amplification for each couple of primers. Click here for file

Additional file 4 **MIQE document (Minimum Information for Publication of Quantitative Real-Time PCR Experiments)**. Click here for file

Additional file 5 **Genes Significantly Expressed in Experiment 2**. For each species analyzed in Experiment 2 (*C. arabica*, *C. canephora*, *C. eugenoides*), genes showing a significant hybridization signal compared to the background noise are indicated. Click here for file

Additional file 6 **Gene Ontology Functional Enrichment**. Functional enrichment using gene ontology terms and Fisher's Exact Test was performed using Blast2GO for the sets of up-regulated unigenes in each of the three tissues (bean, leaf and flower). The full set of unigenes of the PUCE CAFE array was used as the reference set. The resulting p-values are indicated after adjusting for FDR multiple testing results. The over- or under-expressed functions at p ≤ 0.01 are specified. Click here for file

Additional file 7 **Reproducibility of Replicates for the Leaf Tissue in the Leaf-Flower Comparison**. Coefficient of variation (CV%) for all cDNAs spotted on the array based on raw data mean fluorescence values plotted against the relative rank of the CV. CVs were estimated from raw data derived from six replicates (i.e. 2 dye × 3 biological replicates). For this tissue and for this experiment, 90% of the spots on the arrays could be determined with a CV of less than 35%. For the other experiments, 90% of the spots on the arrays could be determined with CV between 32 and 42%. Click here for file

Additional file 8 **Theoretical Power Analysis for a Two-sample T-test to Detect a 1.25 to 2.50 Fold Change in Gene Expression as a Function of CV%**. Indicated sample size = 6 and a Type I error (false positive rate) of 0.1 were used as input values to determine the fraction of changes in gene expression that would be detected at a given CV%. A power of 1.0 denotes a Type II error (false negative rate) of zero, i.e. 100% of all changes that occurred were detected. Click here for file

Additional file 9 **Lists of Genes Specifically Over-expressed in each Tissue Compared to the Two Others (Bean, Flower and Leaf)**. The first 3 datasheets correspond to the lists of genes differentially expressed for each comparison identified by the Limma Analysis (p = 0.01). The last datasheet corresponds to the lists of genes specifically over-expressed in each tissue compared to the two others. Click here for file
