# Supplementary material for: The 'PUCE CAFE' Project: the First 15K Coffee Microarray, a New Tool for Discovering Candidate Genes correlated to Agronomic and Quality Traits
Source: BMC Genomics. 2011 Jan 5;12:5. doi: 10.1186/1471-2164-12-5 (PMC3025959; doi:10.1186/1471-2164-12-5)
Supplement: Additional file 4 — MIQE document (Minimum Information for Publication of Quantitative Real-Time PCR Experiments). [file 1471-2164-12-5-S4.DOC]

| **EXPERIMENTAL DESIGN** |
| --- |
| Definition of experimental and control groups |
| Number within each group |
| **Assay carried out by core lab or investigator's lab?** |
| **Acknowledgement of authors' contributions** |
| **SAMPLE** |
| Description |
| Volume/mass of sample processed |
| **Microdissection or macrodissection** |
| Processing procedure |
| If frozen - how and how quickly? |
| If fixed - with what, how quickly? |
| Sample storage conditions and duration (especially for FFPE samples) |

Experimental design is provided in the material and method section. Tissues (5 g) were harvested in the greenhouse and immediately frozen in liquid nitrogen. Tissues were stored in -80°C freezer until RNA extraction would be performed. Generally, storage did not exceed one week.

Assays (Q-PCR experiment) were carried out by core lab.

| **NUCLEIC ACID EXTRACTION** |
| --- |
| Procedure and/or instrumentation |
| Name of kit and details of any modifications |
| **Source of additional reagents used** |
| Details of DNase or RNAse treatment |
| Contamination assessment (DNA or RNA) |
| Nucleic acid quantification |
| Instrument and method |
| **Purity (A260/A280)** |
| **Yield** |
| RNA integrity method/instrument |
| RIN/RQI or Cq of 3' and 5' transcripts |
| **Electrophoresis traces** |
| Inhibition testing (Cq dilutions, spike or other) |

Tissues were ground into a powder in liquid nitrogen and total RNA was extracted using the RNeasy Plant Mini Kit (Qiagen, Cat# 74904), then treated with RQ1 DNAse (Promega, Cat#M6101) following the manufacturer’s instructions. Total RNAs were finally eluted from the columns with RNase-free water (2x30μL). For each tissue, three independent RNA extractions were performed. All RNA samples were analyzed by formaldehyde agarose gel electrophoresis to assess their integrity.To test for contamination by polyphenols, carbohydrates and proteins, a NanoDrop ND-1000 spectrophotometer (NanoDrop Technologies, DE, USA) was used. Only RNA samples with OD 260/280 > 1.8 and OD 260/230 > 2 were used for further analysis. Exact values for each RNA can be provided upon request. The yield is about 10 μg of RNA per 100 mg of frozen powder.

| **REVERSE TRANSCRIPTION** |
| --- |
| Complete reaction conditions |
| Amount of RNA and reaction volume |
| Priming oligonucleotide (if using GSP) and concentration |
| Reverse transcriptase and concentration |
| Temperature and time |
| **Manufacturer of reagents and catalogue numbers** |
| **Cqs with and without RT** |
| **Storage conditions of cDNA** |

M-MLV Reverse Transcriptase from Invitrogen (Cat#28025-021; 200U/µl) was used for the generation of first strand cDNA in a 20µl reaction volume. 1µg of RNA (variable concentration), 1µL of random hexamer (50µM) and 1µl of dNTP mix (Invitrogen) were mixed, incubated at 65°C for 10min, and quickly chilled on ice. 4µL of 5x Buffer, 2µL of DTT 100mM, and 1µL of RNAsin (40U/uL) were added, and the reaction mix was incubated at 42°C for 2min. 1µL of the M-MLV were added, and the reaction mix was incubated at 42°C for 60min, then at 70°C for 15min. cDNA was stored in low adhesion tubes at -20°C.

| **qPCR TARGET INFORMATION** |
| --- |
| If multiplex, efficiency and LOD of each assay. |
| Sequence accession number |
| **Location of amplicon** |
| Amplicon length |
| *In silico* specificity screen (BLAST, etc) |
| **Pseudogenes, retropseudogenes or other homologs?** |
| **Sequence alignment** |
| **Secondary structure analysis of amplicon** |
| Location of each primer by exon or intron (if applicable) |
| What splice variants are targeted? |

Multiplex qPCR was not performed. Sequence accession numbers and amplicon lengths are included in Additional file 3. Primers were designed in exons or UTR regions close to the 3’end of the gene. No splice variants were targeted.

| **qPCR OLIGONUCLEOTIDES** |
| --- |
| Primer sequences |
| **RTPrimerDB Identification Number** |
| **Probe sequences** |
| Location and identity of any modifications |
| **Manufacturer of oligonucleotides** |
| **Purification method** |

Primer sequences are included in the manuscript as Additional file 3. No modifications were used. Primers were purchased from Eurofins MWG Operon and are salt-free.

| **qPCR PROTOCOL** |
| --- |
| Complete reaction conditions |
| Reaction volume and amount of cDNA/DNA |
| Primer, (probe), Mg++ and dNTP concentrations |
| Polymerase identity and concentration |
| Buffer/kit identity and manufacturer |
| **Exact chemical constitution of the buffer** |
| Additives (SYBR Green I, DMSO, etc.) |
| **Manufacturer of plates/tubes and catalog number** |
| Complete thermocycling parameters |
| **Reaction setup (manual/robotic)** |
| Manufacturer of qPCR instrument |

The PCR reactions were performed using Roche's LC480 and white 384 well-plates. 5 µl of primers at 300 nM were distributed into wells and lyophilized. 2.5 µl of 2x SybrGreen PCR mix (Roche; #4887352001) and 2.5 µl of cDNA at 0.8 ng/µl were then added to each well. PCR parameters were as follow: 10 min. at 95°C, followed by 15 sec. at 95°C and 30 sec. at 95°C for 45 cycles. For the dissociation curves, fluorescence was recorded continuously during a ramp from 60°C to 95°C.

| **qPCR VALIDATION** |
| --- |
| **Evidence of optimisation (from gradients)** |
| Specificity (gel, sequence, melt, or digest) |
| For SYBR Green I, Cq of the NTC |
| Standard curves with slope and y-intercept |
| PCR efficiency calculated from slope |
| r2 of standard curve |
| Linear dynamic range |
| Cq variation at lower limit |
| Evidence for limit of detection |
| If multiplex, efficiency and LOD of each assay. |

The specificity of the amplification products have been confirmed by analyzing their melting curves, by size estimations on a 4% agarose gel, and finally by sequencing of the products. Without a template, no Cq could be determined since it never passed the threshold line. PCR efficiencies are included in Additional file 3.

| **DATA ANALYSIS** |
| --- |
| qPCR analysis program (source, version) |
| Cq method determination |
| Outlier identification and disposition |
| Results of NTCs |
| Justification of number and choice of reference genes |
| Description of normalisation method |
| **Number and concordance of biological replicates** |
| Number and stage (RT or qPCR) of technical replicates |
| Repeatability (intra-assay variation) |
| **Reproducibility (inter-assay variation, %CV)** |
| **Power analysis** |
| Statistical methods for result significance |
| Software (source, version) |
| **Cq or raw data submission using RDML** |

qPCR analysis program (source, version): Roche

Cq’s were determined by setting the threshold to -1.0 using a log scale,

No data have been exclude from the calculations

Results of NTCs: no amplification products present thus no Cqs

Justification of number and choice of reference genes: 3 genes have been selected

Description of normalisation method: endogenous reference gene

Number and concordance of biological replicates: 3

Number and stage (RT or qPCR) of technical replicates: 3 at qPCR level, 1 for RT (Mn samples)

Repeatability (intra-assay variation): was below one Cq
