# Supplementary figures and images for: The 'PUCE CAFE' Project: the First 15K Coffee Microarray, a New Tool for Discovering Candidate Genes correlated to Agronomic and Quality Traits
Source: BMC Genomics. 2011 Jan 5;12:5. doi: 10.1186/1471-2164-12-5 (PMC3025959; doi:10.1186/1471-2164-12-5)

## Slide 1
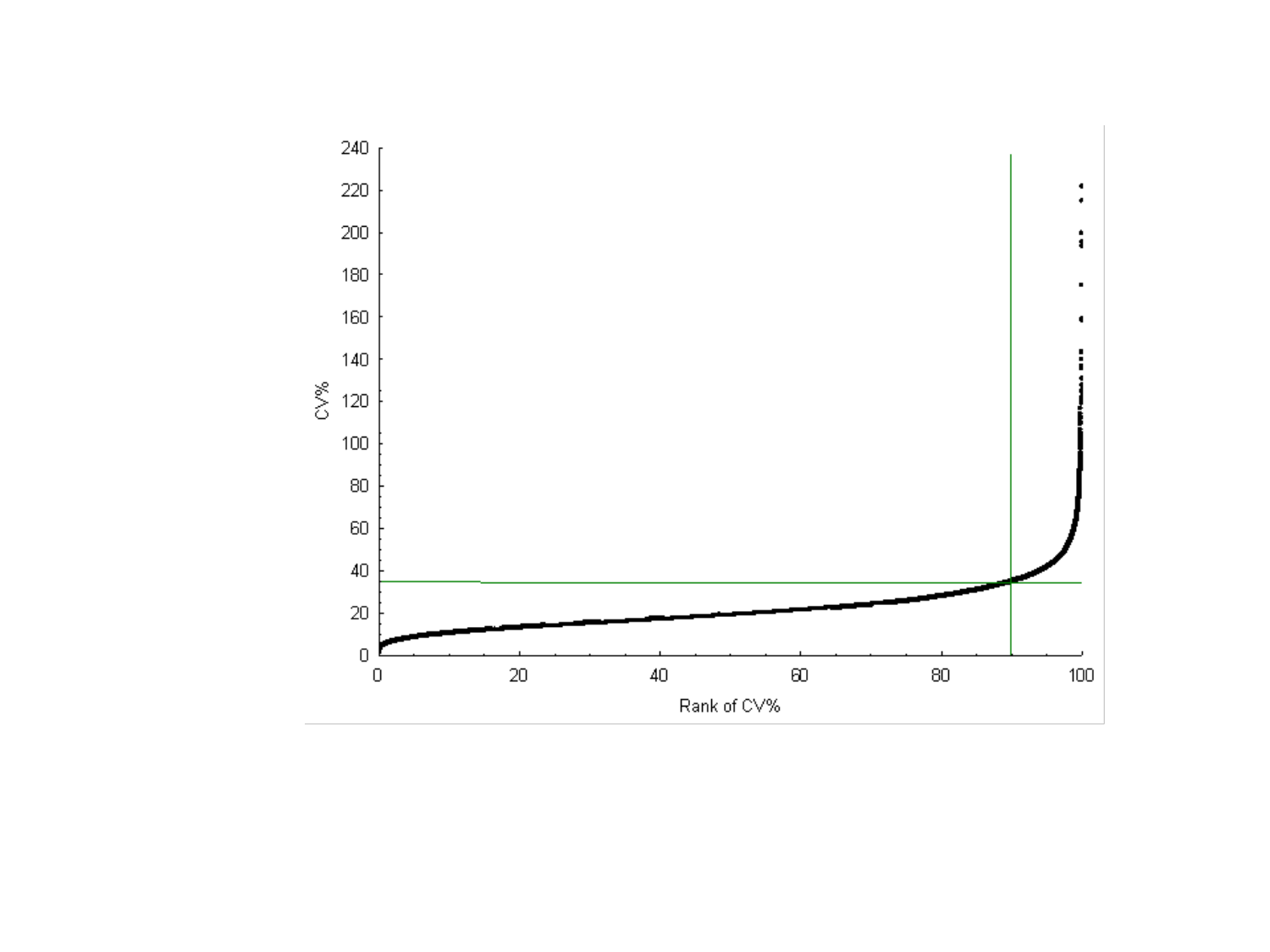

Supplement: Additional file 7 — Reproducibility of Replicates for the Leaf Tissue in the Leaf-Flower Comparison. Coefficient of variation (CV%) for all cDNAs spotted on the array based on raw data mean fluorescence values plotted against the relative rank of the CV. CVs were estimated from raw data derived from six replicates (i.e. 2 dye × 3 biological replicates). For this tissue and for this experiment, 90% of the spots on the arrays could be determined with a CV of less than 35%. For the other experiments, 90% of the spots on the arrays could be determined with CV between 32 and 42%. [file 1471-2164-12-5-S7.PPT]

## Slide 1
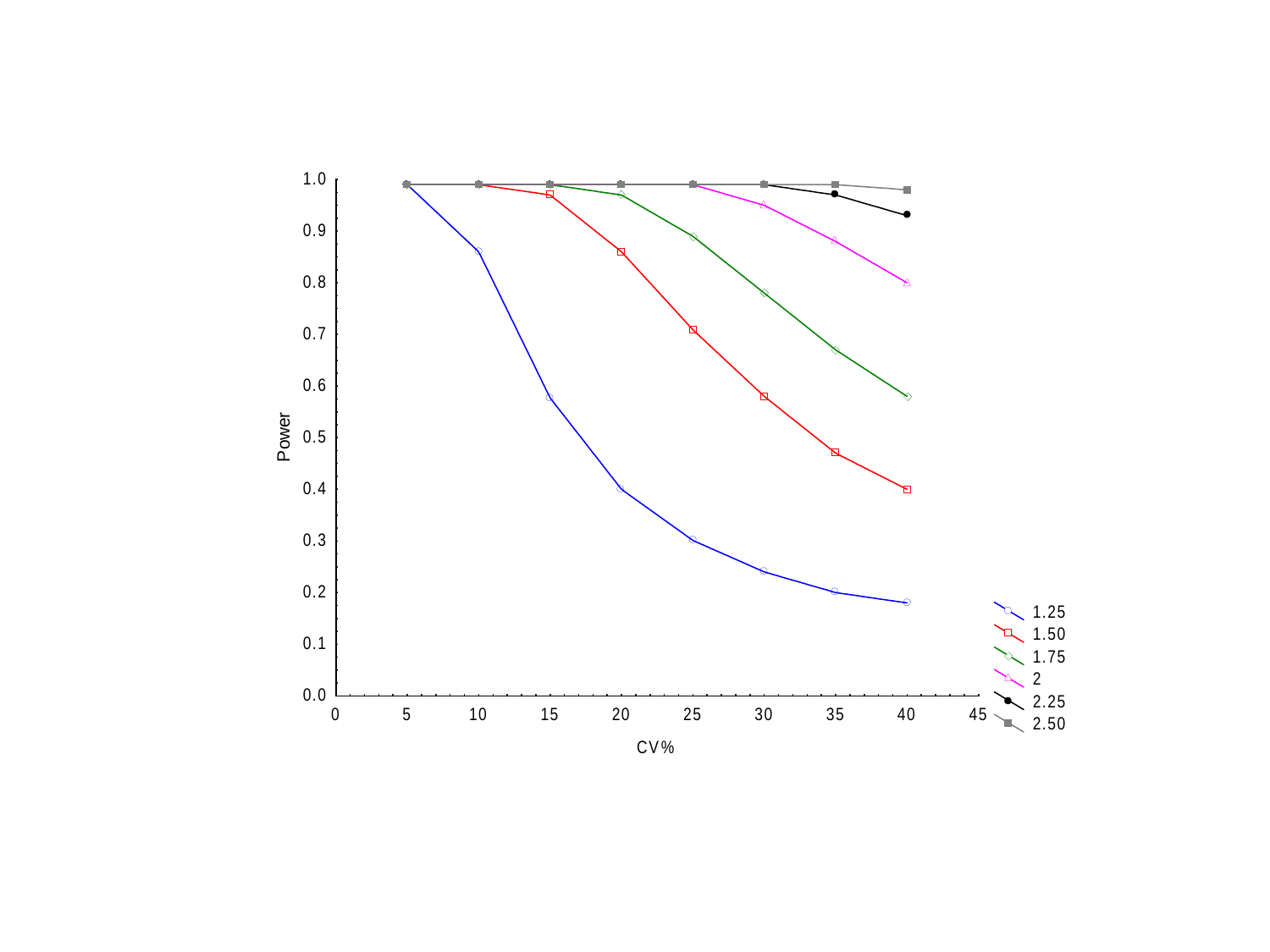

Supplement: Additional file 8 — Theoretical Power Analysis for a Two-sample T-test to Detect a 1.25 to 2.50 Fold Change in Gene Expression as a Function of CV%. Indicated sample size = 6 and a Type I error (false positive rate) of 0.1 were used as input values to determine the fraction of changes in gene expression that would be detected at a given CV%. A power of 1.0 denotes a Type II error (false negative rate) of zero, i.e. 100% of all changes that occurred were detected. [file 1471-2164-12-5-S8.PPT]
